# When Parents Separate and One Parent ‘Comes Out’ as Lesbian, Gay or Bisexual: Sons and Daughters Engage with the Tension that Occurs When Their Family Unit Changes

**DOI:** 10.1371/journal.pone.0145491

**Published:** 2015-12-28

**Authors:** Siobhán C. Daly, Pádriag MacNeela, Kiran M. Sarma

**Affiliations:** School of Psychology, National University of Ireland, Galway, Ireland; Philipps University Marburg, GERMANY

## Abstract

The experiences of Irish sons and daughters born into heterosexually-organised parental partnerships/unions whose parents have separated and one has come out as Lesbian, Gay or Bisexual (LGB) were explored through a grounded theory approach. 15 adult children (over the age of 18 years), who varied in age when their parents separated and one disclosed as LGB, were interviewed. The primary concern that emerged centred on participants having to adjust to their parents’ being separated, as opposed to their parent being LGB. This involved engaging with the tension that arose from the loss of the parental union, which involved changes to the home environment and adapting to new parental partners and family units. Heightened reflection on sexual orientation and an increased sensitivity to societal LGB prejudice were specifically associated with a parent coming out as LGB. How parents negotiated disclosing the changes to others, the level of support available to parents, and how capable parents were at maintaining the parent-child relationship had an impact on the tension experienced by sons and daughters. Participants moved from initially avoiding and resisting the family changes that were occurring to gradual consonance with their altered family environments. Concluding directions for research and clinical considerations are suggested.

## Introduction

This study explores the reflections of sons and daughters who have experienced a parent coming out as Lesbian, Gay or Bisexual (LGB). In contrast to most studies in this area, however, we have sought to probe more broadly the range of experiences that can co-occur with the parent coming out, and in particular relating to the separation and divorce of parents and the impact of this on the child [1, 2].

A central argument underpinning our research is that the coming out of a parent has knock-on effects on other aspects of family life. This can include separation, divorce, a change in residence, and changes in family support. For instance, there is already a developed evidence base relating to the impact of parental separation on children with studies suggesting that experiences relating to changes in parenting, child custody, financial arrangements, extended family relationships, place of residence and daily routine have psychological effects [3]. While the degree of stress arising varies between children, families and over time [4], and the effect sizes are small for long-term negative outcomes [5], some painful emotions such as a sense of loss, anger and anxiety are reported by the majority of children whose parents separate [6, 7].

Having one parent come out can also result in children experiencing stepfamilies headed by both same-sex and heterosexual couples [8], if their parents both form new partnerships and family units. They may also be confronted with societal stigmatisation that can accompany their parents’ homosexuality [9], although this is less of an issue in more progressive urban regions that have LGBT communities [10]. Where this occurs during adolescence, when there tends to be an intense focus on sexual orientation, concerns regarding peer ostracism through being perceived as ‘different’ can be heightened [10, 11].

In contrast to children reared by two LGB parents, older children and adults who experience a parent coming out have to adjust to a change in the sexual orientation of that parent. In adjusting to the reality that ‘my father is gay’, or ‘my mother is a lesbian’, the child or adult must come to terms with the parent having same-gender sexual and romantic relationships [12]. The psychological and emotional well-being of the LGB parents may also play a role here—particularly to the extent that the well-being of the parent influences his/her relationship with the child. A LGB parent who has just come out and their heterosexual spouse or partner may feel isolated, and not have access to relevant support services [13]. This said, in the long term, being out is linked to increased relationship quality between parents and children [14, 15].

Another essential avenue for enquiry is to better understand the factors that improve resilience to negative psychological sequelae of divorce and separation. Research highlights as important the involvement of the non-residential parent [16], diminished parental conflict [17, 18] and parents supporting their children to comfortably maintain relationships with their immediate and extended kin networks [19]. These factors may be particularly important if children have to manage societal LGB prejudice or heterosexism (a cognitive bias that assumes that all individuals are heterosexual in their orientation, and that this is desirable [20]). For example, Bos and colleagues found that a strong child-parent bond helped buffer children against exposure to heterosexism and homophobia [21].

Unfortunately there is dearth of research that has considered the dual impact of separation and the change in sexual identity of a parent on children. Most studies have compared the psychosocial outcomes of children who grew up with heterosexual parents with those who were reared by one or two LGB parents [22, 23, 24], or have focused on children in stepfamilies headed by same sex-couples following a heterosexual separation [25]. Differences in outcomes due to parental sexual orientation have been the primary focus in such studies. The accumulated knowledge of this body of literature, however, does not transfer easily to our understanding of families that were once a heterosexual combined-parent family, but subsequently became a LGB-heterosexual separate-parent family at some point across the lifespan of the child. The changes that occur for both the heterosexual and the LGB parent, and the child’s role in processing these changes is often neglected in favour of a focus on the heterosexual spouse or the LGB parent.

It is against the backdrop of this knowledge vacuum in our area of interest that we decided to explore the experiences of sons and daughters with one heterosexual and one LGB parent. Our aim was to generate an explanatory theory (a model) of how this social experience happens in the context of families. The study was guided by three research questions:

1. How do the children of parents who come out experience and adjust to this change, particularly when it co-occurs with parental separation?
2. What factors influence the adjustment process?
3. Does the age of sons and daughters at the time of separation and disclosure influence their experiences during this time?

## Methods

### Design and Participants

The study had full ethical approval from the National University of Ireland, Galway. All participants signed a consent form and provided informed consent (they were given an information sheet which outlined what their participation would involve and the topic areas that would be explored via interview). Confidentiality was assured as they undertook to retrospectively recount their experience of having a parent ‘come out’ as LGB. Interviews were conducted in Ireland and adapted Grounded Theory techniques were utilised [26, 27]. The resulting theory was “discovered, developed, and provisionally verified” [28] (p. 23) through concurrent data collection and analysis of data relating to the phenomenon of parental separation and a parental disclosure of LGB. To be included in the study participants had to (a) be 18 years of age or older (i.e. an ‘adult child’), (b) have at least one lesbian, gay, or bisexual parent, and (c) have been born into heterosexually organised parental partnerships/unions. LGB parental affirmation of their sexual orientation to the respective adult child must have occurred. Fifteen individuals participated in the study.

Twelve participants were recruited through colleagues and friends of the primary researcher informing people with an LGB parent of the research. Sons and daughters then contacted the researcher directly and expressed an interest in taking part, after receiving more information about the study. Three participants were recruited from the researcher making contacts within LGB associations; two LGB parents passed on the information to their son and daughter, who then contacted the researcher, and a third, a son with a gay father, read an information leaflet published in a LGB magazine and subsequently contacted the researcher.

Data collection ceased when we reached theoretical saturation [29] and new data did not change the core findings that emerged from the data analysis. A profile of each participant is presented in Table 1.

**Table 1:** Participant Demographic Information.

	ID.	Age at time of interview	Age at parental separation	Age at direct informing of parental sexual orientation	Sexual Orientation as defined by sons/daughters	Sexual Orientation ofParents
1	Clare	34	30	30	Straight	Father: Bisexual
						Mother: Heterosexual
2	John	26	11/12	13/14	Straight	Father: Gay
						Mother: Heterosexual
3	Ashton	22	7	7	Straight	Father: Heterosexual
						Mother: Lesbian
4	Betty	52	13	19	Bi-sexual	Father: Gay
						Mother: Heterosexual
5	Barry	19	5	5	Straight	Father: Heterosexual
						Mother: Lesbian
6	Tina	30	Unsure	21	Straight	Father: Gay
						Mother: Heterosexual
7	Andy	28	15	16	Engage in straight relationships	Father: Heterosexual
						Mother: Lesbian
8	Ben	24	13	24	Engage in straight relationships	Father: Gay
						Mother: Heterosexual
9	Sally	30	19 (mother died)	Unsure: 24	Straight	Father: Gay
						Mother: Heterosexual
10	Mark	31	10/11	10/11	Straight	Father: Heterosexual
						Mother: Lesbian
11	Jenny	18	3	3	Straight	Father: Heterosexual
						Mother: “In love with a woman”
12	Tom	19	19	22	Gay	Father: Gay
						Mother: Heterosexual
13	Ann	20	14	14	Straight	Father: Gay
						Mother: Heterosexual
14	Amy	22	6/7	6/7	Bi-sexual	Father: Other/bisexual
						Mother: Lesbian
15	David	21	1	`8/9	80% straight; 20% other	Father: Gay
						Mother: Heterosexual

Eight participants were male and seven were female and they ranged from 18 to 52 years of age (mean age = 26). Data on their age when they were made aware of the change in their parents’ sexual orientation and when their parents separated are provided in Table 1, as are the sexual orientation identification of their parents. Eleven of the 15 were directly disclosed to by their LGB parent (five mothers; six fathers); the remaining four by their heterosexual parent (three mothers; one father).

Five parental unions ended when the child’s father came out as gay and five when their mother came out as lesbian. One father came out following the death of his wife. Two marriages ended when the heterosexual mother had an extramarital affair with a man. One mother began a relationship with a woman after her heterosexual husband had an affair with a woman. One parental marriage remains intact approximately thirty years after the husband disclosed he was gay to his family.

### Data Collection and Analysis

Interviews lasted between 40 and 80 minutes and were digitally recorded. All interviews were semi-structured and focused on when, and how, participants became aware their mother or father was LGB and separating, reactions to the change, changes in family relationships, supportive sources (or lack thereof), experiences of disclosing the parental changes to others, and reflections on sexual orientation in general (self and other). Following each interview the researchers reflected on the core messages that emerged during the interview process, and their context. A ‘funnel-like approach’ [30] (p. 159) postulated by Strauss and Corbin [26] occurred, where the interviewer only moved from broader to more specific questions if specific information did not emerge naturally during the course of the interview.

Each interview was transcribed verbatim and rendered anonymous. Computer assisted qualitative data analysis software (NVivo 9) was utilised to help organise the data and facilitated a more efficient coding retrieval process. As grounded theory requires reflexivity [31], we remained cognisant of the potential influence of our own beliefs and experiences on the data throughout the analytic process. We also drew on past experiences when generating hypotheses, such as the experience of the first author having a father come out as gay following the death of his wife.

Research memos were written, and discussed among the research team in an attempt to reduce potential data bias, and transcripts were reread once the analysis was complete. Epistemological reflexivity occurred by reflecting upon any assumptions made (such as gender based associations or being able to focus on a parent coming out as LGB as removed from the experience of parental separation), and the implications of assumptions on the research process [32].

The analysis/coding protocol utilised was guided by the Grounded Theory coding procedures outlined by Strauss and Corbin, and Corbin and Strauss [26, 27]. The intense and flexible process comprised a three tier coding process involving ‘open’, ‘axial’, and ‘selective’ coding. Data was concurrently collected and analysed, in that the coding process revealed categories and directed further interviews. The process was overlapping and recursive. For example, open coding occurred with respect to new interview transcripts while axial coding was occurring with data from previous transcripts and new data was compared to, and often altered, existing categories.

Open coding involved line-by-line coding of the data/scripts. Key words, phrases and excerpts were assigned names (codes) based on what they represented and grouping of similar codes were collapsed into concepts and renamed. Analytical generalisability, defined as ‘the utility of the concepts to explain a given situation’ [33] (p. 219) was sought. Following the breaking down (multiple categorisation) of the data, axial coding occurred, whereby the categories were refined or clustered into key categories that subsumed several categories. This occurred through making connections or comparisons between categories, supplemented by memo writing and diagramming of the developing processes. For example, when exploring the category ‘indirect disclosure’ we reflected on the nature and context of participant suspicions (such as parental relationship difficulties, parental behavioural changes, and making parental comparisons), what participants did with their suspicions (non-articulation), why they took this action (familial repercussions) and possible consequences. The contextual conditions that resulted in similarities and differences among categories were also focused upon. This process enabled theoretical concepts (for example avoidance and withholding) to be explored in depth.

We adhered to the advice of Corbin and Strauss [27] in allowing the researcher to play a role in analysis and avoided focusing too methodically on analytical procedures to the detriment of the theoretical possibilities contained within the data. Selective coding involved the selection of the core category “family reconfiguration, involving transitional tension”, around which all the other significant sub-categories were integrated and subsumed. The theoretical model was refined through discussions among the authors (which included comparing original transcript excerpts against the emerging theory) to best account for the dynamic processes involved in adjusting to parents as separated and differently orientated, as influenced by family, age and socio-political domains.

## Results

The model below summarises a theory of what happened as the participants in this study processed their parents’ separation and a parent coming out as LGB. It outlines a process of moving from resistance to gradually engaging with the tension that occurs leading up to, and following, the actual disclosure(s). A parent coming out as LGB co-occurred with parental separation and the primary focus of sons and daughters was adjusting to the parental separation, and not the sexual orientation transition of their LGB parent. The process was influenced by how parents dealt with the changes and although common experiences were reported by participants, these also differed according to their age or developmental stage (namely whether they were in childhood, adolescence or adulthood at the time). Although varying degrees of stress were reported, the participants adjusted to the family unit and parental changes that occurred and reported the restoration of consonance (accord) in their family lives.

### Pre-Disclosure: Avoidance

The main factors that participants recalled prior to the disclosure and separation were new parental interests and friendships and in particular acrimony and tension between their parents. While they had a sense that their parents had increasingly diverging identities, they resisted and avoided this reality. Their resistance was characterised by silence and a sense of ‘not wanting to know/believe’, as voicing their suspicions may have signalled the end of their parents’ relationship and changed their family unit. Ann described this as:

*I suspected, but I didn’t say anything to anybody. I didn’t even write it down. It was just an idea but I didn’t want to entertain it because it was just too big to really think about the consequences, of what it would mean for mum and dad*.

Those in adulthood who lived away from the family home did not feel as exposed to the parental difficulties and were less suspicious of the imminent parental separation. Those whose parents were already separated for many years focused more on their parents’ avoidance of dealing with the LGB disclosure at the interview. This is discussed below. Some participants in adolescence and adulthood had subliminal awareness that they only became aware of and “tuned into” after their parents separated. They commented on “signs” such as one of their parents having a ‘new intense friendship’ or in the case of their LGB parent, media coverage which had resonated with them.

### The Disclosure: Parental Processes

The degree of stress and tension reported by participants seemed to be influenced by the parental disclosure process and by the extent to which their parents supported each other and were supported by others.

#### Parental disclosure

Although participants reported feeling varying levels of upset and shock at the disclosure, those whose parents had already separated reported more nonchalant reactions. They felt relieved by the clarity the disclosure provided, or that the disclosure was not about their parent being sick/dying. Intense emotional reactions were reported by the two participants who experienced the separation and LGB disclosure simultaneously (one in adolescence and one in adulthood). Some parents requested that they hold back from informing immediate family members that they or their spouse now identified as LGB. This was identified as stressful by participants. They attributed reasons for the non-disclosure to parental fears that they would be rejected by family members, societal prejudice and/or concern for the well-being of their loved ones. In some instances participants had to be mindful to not let the revealing information accidentally “slip”, which required vigilance. For some it was the LGB parent who encouraged non-disclosure to the other parent, siblings or extended family members, e.g., one young adult’s father came out to him two years before he told his wife, which he described as very isolating and “a lot to carry on his own”. For others it was the heterosexual parent who did not want specific family members, such as younger step-siblings to be directly told. For example Ashton recalled:

*Dad drummed it into us, “you don’t talk to Zara (half-sister) about your mother (being a lesbian)…you’re not allowed talk about it”, but Zara asked us one day and my brother had to say “I’m not allowed talk to you about it but you should ask your mum”. Zara’s really sharp. She just wanted to talk to us about it, to see where we were with the whole thing because no one was broaching it*.

Feeling of guilt occurred if the withholding extended over time, as illustrated by Ben:

*She (mother) still doesn’t know dad’s gay….it’s not my place to tell her. We’ve always been really close, but knowing about my dad and her not knowing, or not sure if she knows, it feels like I’m hiding something from her, it’s that bit of guilt I hold. And I feel that if she knew about dad she might feel a little less guilty that she left*.

Parents who had other family members come out in the past and/or who had LGB friends were perceived as being more comfortable with the disclosure, which was supportive for their children. Overall participants felt that relationships were less strained when their family became more open and comfortable with the separation and sexual orientation changes.

#### Parental boundaries and support

All participants felt that being directly informed of the parental separation and the disclosure of LGB was important and advised parents to recognise that children are perceptive at sensing parental difficulties, as exemplified by Sally:

*I think the way he did it was pretty good. He told us before other people knew. It must have been hard and brave to do but it was important to tell us straight away rather than other people telling us, or finding out some other way. You know when something is bothering your parents*.

The process was made more difficult for participants when they had to manage significant parental acrimony. They reported feelings of discomfort, confusion and anger if their parents (heterosexual/or LGB) made negative comments to them about each other, or ex-spousal partners (mother/fathers new partner). Jenny reported,

*Dad had a very difficult time getting used to it. When I’d be out with him he’d be bad mouthing Maria (mother’s partner) really inappropriately. And I loved Maria. This built up my confusion even more. I thought this is my dad and he doesn’t understand why this is happening so how can I understand why this is happening. I’d then get the feeling of basically wanting to run away, and not wanting to deal with it in any way, shape or form*.

For some parents, their son or daughter was one of a few with whom they could talk to about past relationship or new relationships and intimacy issues. Over time participants began confronting and questioning the appropriateness of being positioned as an intimate confident by their parent(s), as Betty explained,

*Dad started talking more openly to me about different things, like his relationship with another man. Part of me was glad that he’d be talking but at the same time, it doesn’t matter whether you’re gay or straight, you’re my father and I don’t really want to know, and so for a while it became more like a peer thing. I think both my parents shared too much with me; you shouldn’t have to carry that much too early*.

Participants felt relieved when they became aware that others supported their parents, personally through friends/family members and/or professionally, through counselling.

### Post-Disclosure: Tension and Engagement

All the participants reported tension as they tried to accommodate and engage with practical and personal changes that occurred post-disclosure. Family unit changes occurred and the amount of support available to and within the family unit was influential. Three core experiences emerged during the process of accommodation, namely experiences of parental separation (loss), sexual orientation reflection and an increase in LGB sensitisation.

#### Family unit changes

The marital dissolution involved changes to the family unit and home. Sons and daughters who were in adulthood when their parents separated and one came out as LGB were likely to be more independent, living or working outside the family home and caring more for themselves. However, they still experienced changes to their family home, as one parent was now absent, there was a new parental partner in the family home or their parents had sold the family home and moved. They themselves did not have to relocate and could distance themselves somewhat from the process. They were less likely to experience stepsiblings and were more likely to be sought as a support by their parents in processing the separation and the coming out process.

Children in late adolescence or early adulthood continued to have almost equal access to both their parents, whilst younger participants spent most of their formative years with a single parent (with contact with the non-residual parent) or in a stepfamily. Many experienced two stepfamilies, as both their mother and father had subsequent relationships and children, naturally or through fostering/adoption. Sons and daughters who were younger at the time focused more on parental acrimony and the challenge of moving or living between two family homes, as Barry articulated:

*Mum and dad giving out about each other and the practicality of having to go from one house to the other every week was the most difficult thing…I always left something behind, school books, school uniforms. You’d have to have two sets of everything!*

#### Protective support

Not feeling isolated or “alone” during the family changes was identified as important. Participants who felt they had significant support from their mother or father during the separation and disclosure process reported a reduced need for additional support(s). Those who had access to sibling support found that humorous interactions helped defuse family tension arising at family occasions. Relief and reassurance was also gleaned from talking with open minded friends who supported sexual and familial diversity. For older participants this meant some changes to friendships already in place, whereas younger participants usually only formed friendships with those perceived to be “open minded”.

Whilst many participants had peers whose parents had separated or divorced, they were ‘tuned into the fact’ (largely as a result of the reactions of others) that having a parent come out as LGB later in life seemed more unique. Becoming naturally aware that their family situation was far more common than they had first realised was named as supportive. Those who were younger when the family changes occurred had more access to support in this regard; many had spent time in the company of other children with same sex parents or one LGB parent. Older adolescents and adults were less likely to know other sons or daughters with a LGB parent. They reported that until participating in the study they had not met another who had a parent come out later in life.

Some participants accessed professional support to manage difficult thoughts and feelings which resulted from significant parental acrimony. Others did so as a result of being the only family member disclosed to by their LGB parent for a prolonged period of time, or from feeling isolated in general. Such support was identified as helpful and needed. Support from their extended family made the family changes easier and seemed to reinforce a sense of normality and acceptance that the participants were seeking, as outlined by Bernie:

*The attitude of my family helped me the most. They made me feel that this is ok, this is normality; this is just the way it is. Nobody in the family pulled me aside and said “this is not right,” “you’re different from everyone else”. Everyone carrying on as normal was great because I didn’t at that time want to stand out, I wanted to blend in*.

Participants felt protective of their parents and angry with extended family members (grandparents, aunts, uncles) if they were perceived to ‘reject' or ‘blame’ their parent for breaking up the family unit (through making negative statements, ceasing communication, or metaphorical acts such as removing family photographs from walls). They also tended to avoid such family members where possible or were vigilant in the information they shared with them.

#### Mourning the parental separation

Participants perceived their parents as being fundamentally “the same” before and following the separation and disclosure, irrespective of their sexual orientation. However, participants still had to adjust to their parents being separate and single. Mourning the loss of the parental union was experienced by participants for several years after their parents parted. For some additional loss also occurred when parents repartnered and these relationships subsequently broke up after many years, or a new parental partner died.

Although participants reported some initial discomfort in seeing their parents (both heterosexual and LGB) with new partners, they wanted their parents to have appropriate parental partnerships or friendships so they would not feel sad or lonely. For very young children parental repartnering was less of an accommodation, as they had no or limited memories of their parents as single. Seeing a parent form new relationships was more of an adjustment for participants who were near or in adulthood, after a lifetime of seeing their parents as a couple. The word “awkward” was frequently used when recalling meeting new parental partners. They expressed concerned for their parents’ mental and physical health, and sadness in seeing their parents adjust to the separation and to being single. Adult sons and daughters had had more collective and parental memories to explore and reflect on. Some reported feelings of resistance to looking at old family photographs, as expressed by Tina:

*I feel sadness looking back at family photos and seeing us all together as a family with mum and dad and pictures of their honeymoon or their wedding. I’m really sad just for both of them*.

Sadness in having to question their parents’ marriage was coupled with the belief that “they were good memories, and real moments” (Clare).

Participants empathised with their parent in coming out later in life. Many felt a lack of family or religious acceptance formed the root of their parent not coming out earlier, while some felt the decision of their parent’s disclosure was unintended or accidental, but inevitable.

Participants expressed varying degrees of anger towards the parent who was perceived to initiate the family break-up (heterosexual or LGB) through having an affair, or leaving. Sadness at the family dissolution was coupled with a perception that their parents would not have been fully satisfied if they had remained together, as exemplified by Ann:

*I think most children of broken homes or spilt-up families would think things like “it would be pretty good if they were together”, but it also wouldn’t have been good, cause they would have been unhappy. Even if they weren’t shouting and screaming there’d be tension and something unfulfilled*.

The participants in the main avoided asking their parents if they had had an extramarital affair, or when their LGB parent knew they were LGB. For some this was unarticulated due to the possible implications this could have on their memories of the parental union, and the heterosexual parent, but in general the topic was avoided as participants did not want to reflect on their parents’ sex lives, as illustrated by Andy,

*I don’t really want to think about or explore my parent’s sexual past, or present! Who does?!*

#### Sexual orientation reflection

Having a parent come out as LGB resulted in enhanced reflection on sexual orientation differences. This, in addition to an increased awareness of LGB prejudice, was unique to the process of a parent coming out. Following the disclosure, adult offspring wondered about the sexual orientation of others, and of potential partners. Participants who went through adolescence following the disclosure reflected more on their own sexual orientation. They recalled feeling some confusion and curiosity regarding their orientation, especially as their parents differed in this regard. Participants who were very young when their parent came out tended to devote less focused thought on the sexual orientation of others. Differing sexual orientation was nothing novel. For example, Mark reported,

*People’s sexuality is such a non-issue for me because I grew up around homosexuality….It’s never really held any mystery for me, I mean, not to trivialise it, but to me it’s like do people like tea or coffee*.

As participants became increasingly comfortable with sexual orientation differences they reported spending spent less time focused on sexual orientation in general.

#### LGB prejudice sensitisation

Having an LGB parent resulted in an increased awareness of societal prejudice, including heterosexism and homophobia for all participants. Although they made others aware that their parents had separated with little reservation, they tended to be more cautions and sometimes avoided informing others of the LGB parental orientation. They found the reactions of others to the disclosure supportive in general, however, vigilance occurred regarding what was “acceptable” in their social environment and social situations were often assessed before decisions regarding disclosure were made. Many limited the information they shared with those who may have viewed the orientation of their LGB parent as bizarre, shocking or problematic. Limited disclosure occurred particularly for participants within their work environments and during their school age years where they felt more pressure to ‘blend in’. David explained:

*It wasn’t that I was ashamed, I’m very proud of my dad. It was more that I knew that it wouldn’t fly because I came from the country where some people can be very narrow minded…so if I’d told anyone in school they would have been like, what, that’s bizarre. It might have affected me and dad socially*.

No participant wanted their parent to be treated or judged differently due to their orientation. They reported feeling ‘protective’ and ‘proud’ of both their parents as a result of societal LGB prejudice. References were made to being attuned to heterosexist or homophobic comments and a sense of resulting frustration emerged at times. For example, Amy commented:

*I’m quite protective of mum and I get really, really annoyed when people are in any way homophobic. I wouldn’t be mean or feel attacked. But I’d feel annoyed with that person and not want anything to do with them*.

Feelings of concern were also expressed towards their parents’ new partners if they did not seem to have their parent’s best interests at heart, e.g., were perceived as “gold diggers” or were “taking advantage” of their parent(s). This level of worry decreased over time, as participants saw supportive parental relationships develop. They found it increasingly difficult to tolerate heterosexist attitudes from friends or colleagues, and distanced themselves from, or avoided such company or sometimes engaged in ‘educational’ dialogue or debates, striving for balance in this regard, as exemplified by Ashton:

*Now I find I’m confident enough that if somebody would have a problem I would challenge them, you know not in a nasty way….if somebody said you know gay people shouldn’t be allowed to have children or whatever, I would try and give another point of view, or I’ll remove myself from the situation*.

With reference to the culture in which this study took place, which has been a traditional one [34], participants believed that Irish society has become more tolerant of sexual orientation differences and sexual fluidity in recent years. They felt this has made their life and the life of their LGB parent “easier”. While older participants were more fixed in defining their sexual orientation as “definitely” heterosexual or gay, sexual orientation was viewed more as a fluid concept by younger participants, as articulated by Andy:

*I’ve tended to think more in terms of people being fluid. I think society is the thing that imposes us as being one thing or another. I don’t think anyone can be completely 100% straight; ultimately you never know at the end of the day. There could be one person of the same sex that you just find irresistible!*

### Consonance

Positive outcomes and relationship healing between parents, extended family members and child-parent relationships was reported by the majority of participants. In the few cases where parental acrimony and family difficulties continued to exist, participants felt that they were able to manage on-going issues with greater understanding and confidence, and at a greater distance. Mark surmised:

*I feel a lot of love for both my parents. I used to get angry, but after everything that’s happened, they love me. I’ve given it enough energy. I’m more interested in building my life and looking forward*.

All the participants acknowledged the importance of support that was available naturally to them, as Ann highlighted:

*Talking naturally with friends and family helped….you don’t want it to be like an AA group sitting around, “Hi I’m Ann, my parents have broken up, dad’s gay!”*

Due to the retrospective nature of the study many references were made to the passage of time (“time to adjust”; “transition time”, “time as a healer”) in accommodating changes and in making what was new familiar. Time seemed to allow participants and the significant persons in their lives to become increasing comfortable with altered family reconfigurations. Participants reported feelings of love and pride towards their parents, who exist in a society where they feel some prejudice towards LGB persons remains. Although the transition from the ‘traditional’ family unit to having blended family unit(s) and an LGB parent was a difficult process, involving additional, and for some continuing challenges of varying degrees, overall consonance occurred.

## Discussion

**Fig 1:**
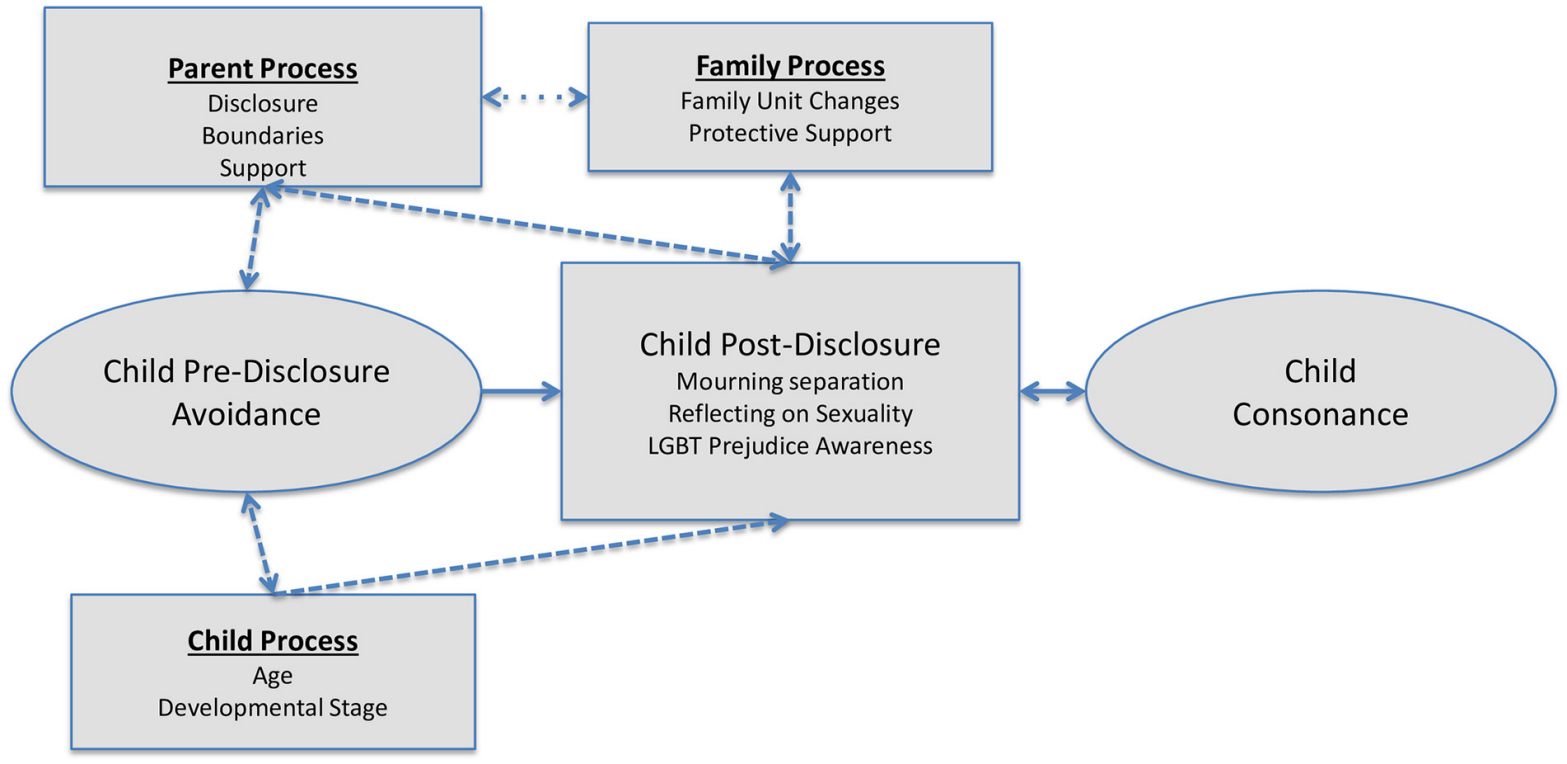
Sons and daughters engaging with tension that arises (when parents separate and one comes out as LGB): A transition from avoidance to consonance.

The conceptual model we developed (see Fig 1) represents a process of adjustment by sons and daughters who have experienced a parental separation and a parent coming out as LGB. Regardless of age, the primary concern for participants centred on the transition from the nuclear family to separated parents and blended family units. The model outlines the movement from wanting to avoid family unit change to engaging with the loss of the parental unit (involving altered family environments and parental relationships) and finally attaining consonance in having separated and differently sexually orientated parents. The theory arising from this study has similarities with previous grounded theory studies which highlight contextual factors and loss in post-divorce families [35] and the importance of positive parent-child communication in family adjustment [35,36]. However, unlike the previous studies, our model also incorporates the experience of a parent coming out as LGB.

An increase in tension was shown to occur for sons and daughters who experienced significant parental acrimony or isolation arising from parental non-disclosure (where parents disclosed to the participant rather than their marriage partner or siblings). Participants strove to reduce the tension that arose through strategies such as empathy, confrontation and resistance, which is in keeping with the literature on tension or dissonance aversion [37,38]. The experiences specifically associated with a parent coming out, namely increased sensitivity to societal LGB prejudice and reflection on sexual orientation, although minor, caused additional sources of tension at times. The ‘unusualness’ of a parent coming out seemed influenced by participant, family and community exposure to, and acceptance of, LGB diversity. In general, overall adjustment was reported by participants following the family unit and parental changes.

Our findings suggest that the manner in which parents and the family as a whole manage the changes and the support that they receive have an influence on the adjustment process of sons and daughters. This is comparable to other studies which highlight the link between how a family as a whole adapts to change and adjustment in young people [39]. As expected protective factors, such as positive parental support, reduced parental conflict and comfortably maintained child-parent and extended kin relationships [16, 19, 36] facilitated a smoother transition for sons and daughters during the adjustment process. These factors seem even more important where societal sexual prejudice exists. Parents who come out later in life, and their spouses, are less likely to be involved in LGB or heterosexual (straight spouse, for example) family support organisations or to know others in similar situations [13]. It may be that feelings of parental protectiveness evoked by LGB prejudice strengthens the child-parent relationship, and possibly enhances resilience through this connection. Clearly, enabling family members to access support where required is important in family adjustment, as situations in which individuals perceive themselves as being ‘alone’ in processing a societal stigma can lead to negative psychological consequences [40], including depression and anxiety [41, 42].

We showed that parental non-disclosure to others resulted in vigilance and isolation, whilst parental openness enhanced child-parent communication. Immediate and extended family disclosure and support impacted positively on the family adjustment as a whole, which supports the association between disclosure or “outness”, positive self-acceptance [20] and enhanced child-parent relationship quality [14, 15]. Our findings suggest that the ability of sons and daughters to avail of, and access parental and family support decreases when they feel unable to tell significant family members that their mother or father is LGB. This may result in a greater need for professional support as a consequence of withholding information from others.

Our findings support the premise that the age and developmental stage of sons and daughters at the time of separation and disclosure can result in experiential differences. For example, we found that sexual self-questioning was more salient for participants during adolescence (which is in keeping with the literature [43]), whereas older, adult children reflected more on the orientation of others following a parental disclosure of LGB. Mourning the loss of the parental union was also more intense for older participants, as they had more memories of their parents as a couple. They were also more likely to be a source of support to their parents than their younger counterparts. The current study conflates and combines several research questions which may be better explored singly, such as possible age related differences for sons and daughters when a parent comes out before or during or after separating from their partner. Recruiting larger samples of sons and daughters and exploring their experiences at differing developmental stages, including adulthood, may add additional depth to the current findings.

An important contribution of the current work is the finding that the process of parental separation and a parent coming out as LGB has different trajectories. Some parental relationships ended as a result of either the heterosexual or the LGB parent having an affair, or as a result of a spousal death. Some unions ceased when a spouse came out as LGB; some parents were aware that they differed in their sexual orientation before they married, but sought other relationships over time. Many transitioned into stepfamily units following the disclosure. While the literature is substantial on stepfamilies [3, 43, 44], it can be disjointed in focusing more on the transition from nuclear to gay or lesbian stepfamilies or LGB single families. It would appear that consideration needs to be given to the fact that children whose parents separate and one parent comes out often experience both gay or lesbian stepfamilies and heterosexual stepfamilies. Educators and therapists should be mindful of any societal prejudice which family members may experience (as social stigma can accompany gay stepfamilies [45]), and be cognisant of the variety of family forms and altering sexual orientation(s) that can occur, uniquely, over time.

The limitations of this study are acknowledged. Our insights and conclusions are drawn from participants in Ireland who were in a position to speak about their experiences, and who had generally adjusted positively to their family changes. The study excluded the children of Transgender parents, which should be included in further studies. Furthermore our sample size was small. However, we reached theoretical saturation quickly as we analysed and collected the data in tandem [29]. Despite the variability of ages and family backgrounds the participant reports showed high convergence with each other and an explanatory theory of the social processes involved emerged. Although the small sample size and methodology prohibits the generalisation of findings, replicability is not relevant given that the focus of theory generation is to offer a new perspective on a given situation which may guide further exploration and focus [46]. While the findings need to be interpreted with caution given the respective nature of the data (which can be influenced by memory bias or how participants felt at the time of interview), the reflective accounts enabled the formation of, and insight into, an experiential process of adjustment.

In conclusion, some sons and daughters in Ireland experience both parental separation and a mother or father disclosing a change in his or her sexual orientation. This involves varying degrees of tension as the family unit changes and associated processes are accommodated. Where support is sought, the model developed herein should help clinicians and educators have a better understanding of the factors which can exacerbate or reduce familial stress, and to respond sensitively to the complexities inherent in this journey [47,48].
