# Correction: When Parents Separate and One Parent ‘Comes Out’ as Lesbian, Gay or Bisexual: Sons and Daughters Engage with the Tension that Occurs When Their Family Unit Changes

**DOI:** 10.1371/journal.pone.0151120

**Published:** 2016-03-02

**Authors:** Siobhán C. Daly, Pádraig MacNeela, Kiran M. Sarma

The second author’s name is spelled incorrectly. The correct name is: Pádraig MacNeela.
